# BRCA1/2 mutations perturb telomere biology: characterization of structural and functional abnormalities *in vitro* and *in vivo*

**DOI:** 10.18632/oncotarget.5693

**Published:** 2015-10-16

**Authors:** Orit Uziel, Rinat Yerushalmi, Lital Zuriano, Shaden Naser, Einat Beery, Jardena Nordenberg, Ido Lubin, Yonatan Adel, Daniel Shepshelovich, Hagai Yavin, Irit Ben Aharon, Shlomit Pery, Shulamit Rizel, Metsada Pasmanik-Chor, Dan Frumkin, Meir Lahav

**Affiliations:** ^1^ The Felsenstein Medical Research Center, Beilinson Medical Center, Tel-Aviv University, Tel-Aviv, Israel; ^2^ Sackler School of Medicine, Tel-Aviv University, Tel-Aviv, Israel; ^3^ Institute of Oncology, Davidoff Cancer Center, Beilinson Medical Center, Tel-Aviv University, Tel-Aviv, Israel; ^4^ Bioinformatics Unit, Faculty of Life Sciences, Tel-Aviv University, Tel-Aviv, Israel; ^5^ Nucleix Ltd. Tel Aviv, Petah Tikva, Israel; ^6^ Internal Medicine A, Beilinson Medical Center, Petah Tikva, Israel; ^7^ Institute of Hematology, Davidoff Cancer Center, Beilinson Medical Center, Petah Tikva, Israel

**Keywords:** telomeres, BRCA1/2, malignant transformation, telomere homeostasis

## Abstract

BRCA1 mutation is associated with carcinogenesis, especially of breast tissue. Telomere maintenance is crucial for malignant transformation. Being a part of the DNA repair machinery, BRCA1 may be implicated in telomere biology. We explored the role of BRCA1 in telomere maintenance in lymphocytes of BRCA1/2 mutation carriers and in *in vitro* system by knocking down its expression in non-malignant breast epithelial cells.

The results in both systems were similar. BRCA1/2 mutation caused perturbation of telomere homeostasis, shortening of the single stranded telomere overhang and increased the intercellular telomere length variability as well as the number of telomere free chromosomal ends and telomeric circles. These changes resulted in an increased DNA damage status. Telomerase activity, inducibility and expression remained unchanged. BRCA1 mutation resulted also in changes in the binding of shelterin proteins to telomeres. DNMT-1 levels were markedly reduced both in the carriers and in *in vitro* system. The methylation pattern of the sub-telomeric regions in carriers suggested hypomethylation in chromosome 10. The expression of a distinct set of genes was also changed, some of which may relate to pre-disposition to malignancy.

These results show that BRCA gene products have a role in telomere length homeostasis. It is plausible that these perturbations contribute to malignant transformation in BRCA mutants.

## INTRODUCTION

Breast cancer accounts for 22.9% of all cancers in women [1]. Mutations in BRCA1/2 genes are identified in approximately 10% of breast cancer patients. Harboring BRCA1 mutation is associated with 43%-75% lifetime risk for breast or ovarian cancer [2–4] and with increased incidence of other malignancies [5]. The mechanism of BRCA1 associated malignancy is unclear. BRCA1 is involved in DNA damage repair by homologous recombination (HR) and non-homologous end joining (NHEJ). Recently it has been shown that BRCA1 dictates the type of DNA repair mechanism at double strand breaks (DSB) together with p53. The interaction of BRCA1 and p53 may increase DNA sensitivity to damaging agents as cytotoxic drugs and radiation [6]. BRCA2 is the Fanconi anemia D1 protein [7] and plays a key role in DNA repair [8–12] and control of genome integrity [13]. Furthermore, BRCA2 has a role in cell proliferation processes.

Telomeres are DNA elements found at the ends of linear chromosomes consisting of hexameric TTAGGG repeats and terminating in single stranded overhang forming a T-loop structure. Telomeres are protected from being recognized as DSB by a complex of six proteins termed “shelterin” (TRF1, TRF2, TIN2, TPP1, POT1, and RAP1), which contribute to the formation of the T-loop forming a “cap” at telomere ends, thus inhibiting the activity of NHEJ and HR [14–16]. Due to the “end replication problem” telomeres shorten with each cell division. Upon reaching a critical length the cells stop dividing and enter senescence. This physiological shortening of telomeres is regarded as an anti-cancer mechanism since it limits the replicative potential of the cell. Damaged cellular checkpoints will result in further cell divisions and telomere shortening leading to telomere dysfunction causing DNA damage and eventually resulting in death or malignant transformation [17]. Telomeres become dysfunctional when their single stranded overhang becomes too short [18]. Telomere length is tightly regulated by a complex homeostatic mechanism, which includes telomerase activity, the shelterin complex and the methylation status of the subtelomeric region. Shelterin proteins are negative regulators of telomere length; some through inhibition of telomerase recruitment or its access to telomeres and others through yet unknown mechanisms [14]. Recent evidence indicates that epigenetic modification of subtelomeric chromatin influences the regulation of telomere length [19]. Several subtelomeric regions have a high density of CpG islands which are susceptible to DNA methylation [20, 21]. In DNMT-1-deficient cells demethylation of subtelomeric regions induce telomere elongation [22]. Telomere shortening can be attenuated by telomerase, a reverse transcriptase that elongates telomeres from 3′ to 5′ [23]. Telomerase activity is suppressed in most human somatic cells [24] and retained only in germinal cells and to some extent in stem cells.

Active telomerase is a hallmark of cancer cells, found in >90% of all tumor types [17]. Telomerase up regulation is required for cancer cell perpetuation and immortality [25].

Several studies reported on possible interactions between BRCA1/2 and the telomere-telomerase system in cancer cells. Overexpression of BRCA1 inhibited telomerase activity by the inhibition of c-Myc binding to hTERT promoter [26]. BRCA1 knockdown increased telomerase activity and the length of telomeres in cancer cells [27]. BRCA1 overexpression caused shortening of telomeres in several cancer cell lines, independent of telomerase activity [26]. Another study showed that BRCA1 knockdown lead to chromosomal instability due to telomere dysfunction [28]. BRCA1 was shown to be bound to telomeres through interactions with TRF1 and TRF2 mediated by RAD50. Furthermore, BRCA1 affects the length of the telomeric 3′ overhang. The mechanism underlying telomere shortening by BRCA1 is still elusive. Inhibition of telomerase by BRCA1 may represent tumor suppressor function activity [29].

We surmised that BRCA1/2 mutations may induce perturbations in telomere-telomerase system and thus contribute to the process of carcinogenesis. The literature regarding this issue is based mainly on data from cancer cells. We assessed telomere-telomerase dynamics in nonmalignant cells of BRCA1/2 mutation carriers. To validate our findings we also created an *in vitro* system in which BRCA1 was silenced in breast epithelial cells to study the effect of this knockdown on telomeres and telomerase biology.

## RESULTS

### Telomere dynamics is different in BRCA1/2 mutations carriers and after BRCA1 silencing

**Figure 1:**
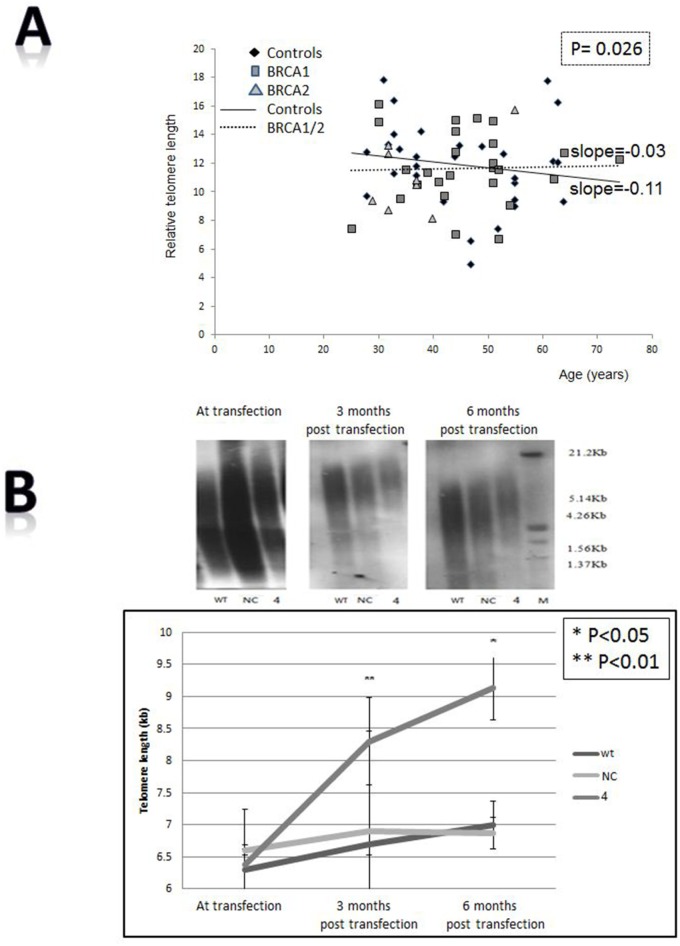

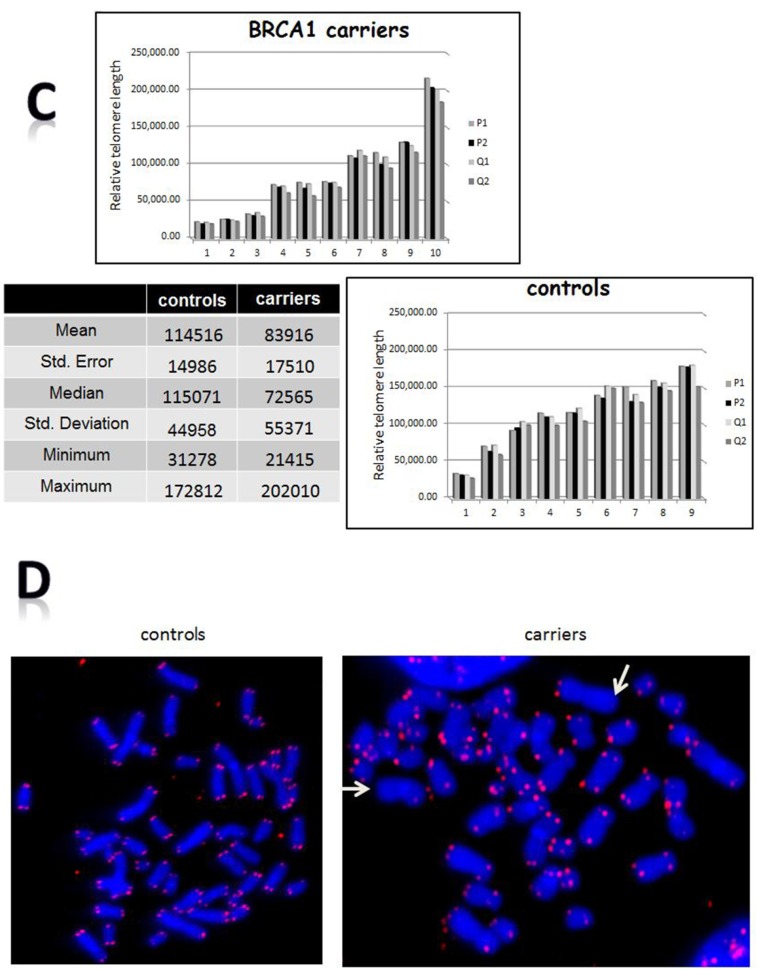
Effects of BRCA mutation on telomere length characteristics **A.** Telomere length in BRCA1/2 carriers. The length of telomeres was measured in BRCA1/2 mutations carriers and compared to healthy controls at similar age groups, by the flow FISH method. The slope of each group is depicted. Since there was no difference between the carriers of BRCA1 mutation and BRCA2 mutation, we draw the regression line for the mutant carriers as a single group. **B.** Elongation of telomeres induced by BRCA1 silencing. Telomere length of HB-2, breast cancer epithelial cells was measured by Southern blot before and after the silencing of the bRCA1 gene. The upper panel shows an example of three blots: before shRNA of BRCA1 gene and three and six months post transfection. “WT” are the wild type cells; “NC” cells transfected with the negative control shRNA plasmid; “4” is clone # 4 in which BRCA1 silencing was optimal 6 months post shRNA transfection. The lower panel describes a quantitation of three independent telomere measurements in two independent knockdown experiments. Effects of BRCA mutation on telomere length characteristics. **C.** Telomere length of different chromosomal arms as measured by Q-FISH in 10 BRCA1 mutation carriers and 9 normal controls. The upper graph describes the control group whereas the lower describes the carriers group. The table depicts the various statistical variants calculated from the FISH data. A more detailed table containing the full data with regards to each chromosomal arm is in the supplemental material. **D.** An example of telomere length analysis by Q-FISH in BRCA1/2 carriers. Arrows point to telomere free ends chromosomal arms which are increased in the carriers. Numbers of chromosomes with no telomere signals are depicted in the text.

Figure 1A shows the telomere lengths of 84 BRCA1/2 carriers and 64 controls. As opposed to the control group in which telomeres physiologically shortened with age, the telomeres of BRCA1/2 carriers did not shorten implying a defective regulation of telomere length. These findings were statistically significant according to T test in which dummy variable of group interaction between age and group was used (P = 0.026). The difference in telomere dynamics between these two groups is also reflected in the slopes of their regression lines; whereas the slope of the control regression line group is -0.11 (describing the expected negative correlation between age and telomere length in healthy population), that of the carriers is - 0.03. Notably, there was no significant difference in telomere dynamics between the BRCA1 and BRCA2 carriers. *In vitro* the telomeres of silenced cells became gradually longer as shown in Figure 1B. In order to verify that in terms of telomere dynamics the silenced cells represent BRCA1 mutation, we measured telomere length also in BRCA1 mutated HCC1937 cells. Indeed, telomeres of these cells were significantly longer than those of wild type breast HB-2 cells (6.5 kb vs. 11kb, Supplementary Figure S2). In Q-FISH analysis of single chromatids the telomere length distribution in carriers is wider than in controls (Figure 1C, Supplemental Table S1). The mean and median lengths are lower in carriers but their variability is higher as manifested by higher SD value (small Table within Figure 1C). These differences did not reach statistical significance. However, the analysis showed a statistically significant higher incidence of telomere free ends in the carriers’ chromosomes. Of 9200 chromatid ends of carriers 331 were telomere free versus 116 of 8280 chromatid ends of controls (3.6% versus 1.4%) implying loss of genomic stability in this group (Figure 1D).

### BRCA1 silencing does not affect telomerase activity or expression

**Figure 2:**
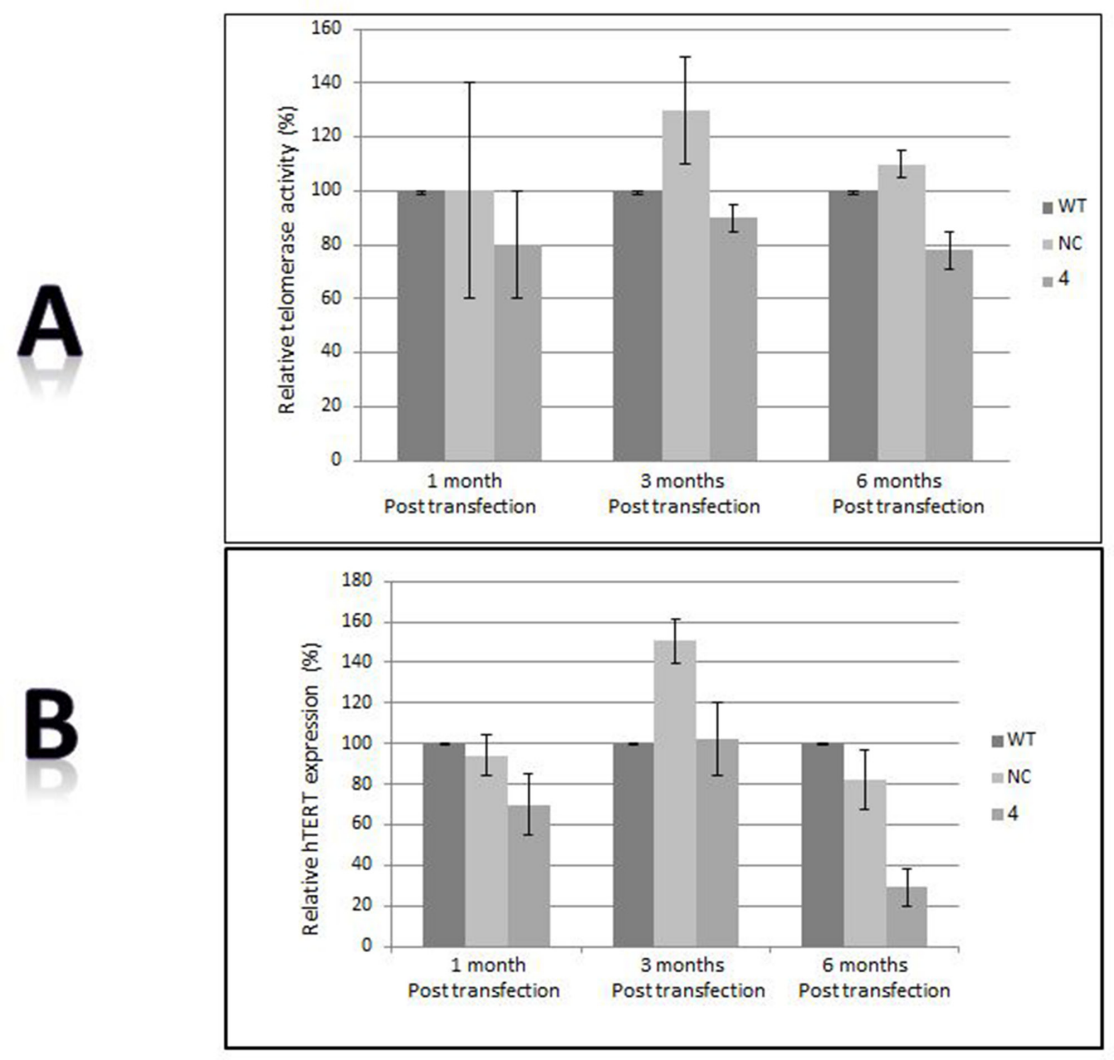

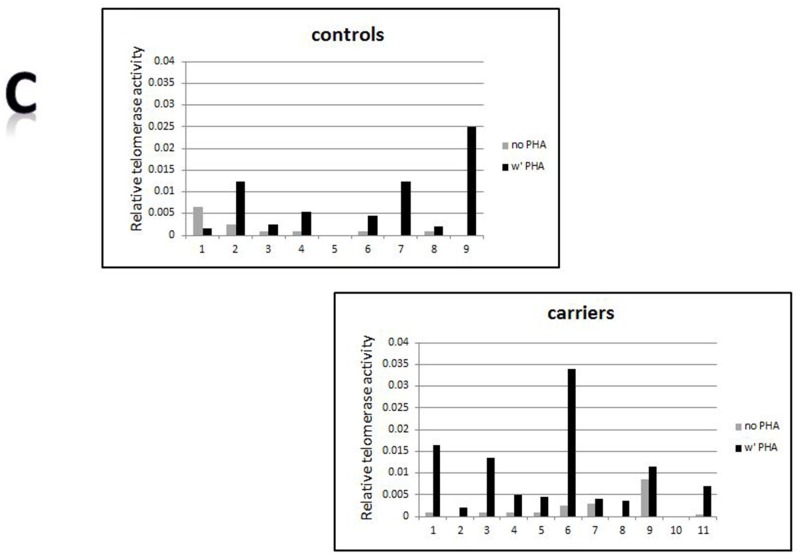
Telomerase involvement **A.** Telomerase activity as measured by the TRAP assay before and after BRCA1 silencing. **B.** hTERT expression measured by real time PCR before and after BRCA1 silencing. “WT”- the wild type cells; “NC”- cells transfected with the negative control shRNA plasmid; “4” is clone # 4 in which BRCA1 silencing was optimal 6 months post shRNA transfection. **C.** Induction of telomerase activity by PHA in BRCA1/2 mutations carriers. Mononuclear cells were isolated from the BRCA1/2 mutation carriers as well as from the control group and subjected to PHA. Subsequently, telomerase activity was measured by the TRAP assay.

Telomerase activity is a major regulator of telomere length. BRCA1 silencing did not alter telomerase activity or expression in BRCA1 silenced cells (Figure 2A, 2B). Normal peripheral mononuclear cells express only minimal or negligible telomerase activity. The telomerase activity of these cells in the carriers was also negligible, similarly to normal controls. Telomerase activity can be induced by *ex-vivo* exposure to phytohemagglutinine (PHA). The degree of induction of telomerase activity did not differ between BRCA1/2 carriers and controls (Figure 2C).

### The levels of γH2AX are higher In BRCA1/2 mutations carriers and *in vitro*

**Figure 3:**
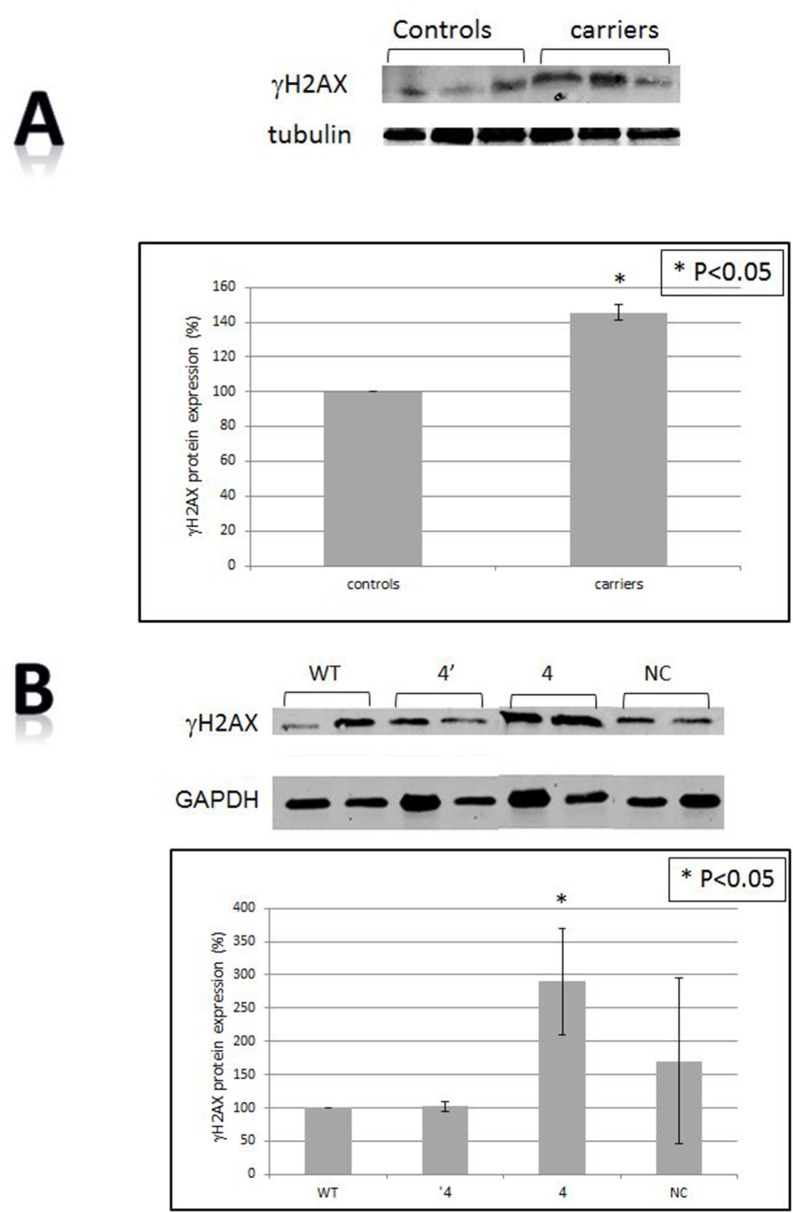

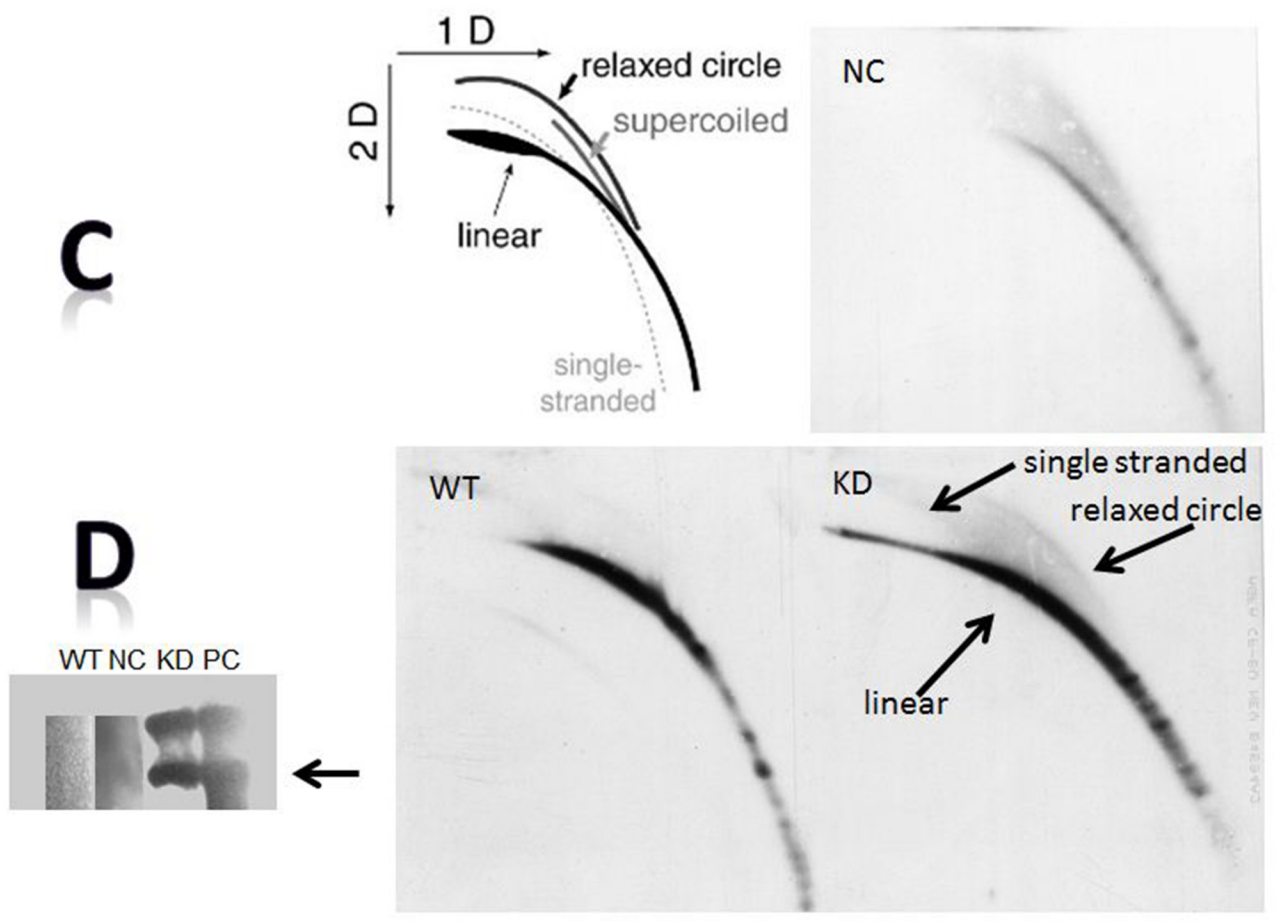
DNA damage **A.** The levels of γH2AX in BRCA1/2 mutations carriers by Western blot. The upper panel is an example of a Western blot and the lower one presents quantitation of three independent measurements. Calculations were done by using tubulin signal as the reference protein. **B.** The levels of γH2AX after BRCA1 silencing as measured by Western blot. The upper panel is an example of a Western blot and the lower one is a quantitation of three independent measurements. Calculations were done by using the GAPDH signal as the reference protein. “WT”- the wild type cells; “NC”- cells transfected with the negative control shRNA plasmid; “4’ “- clone # 4 in which BRCA1 silencing was optimal one month post shRNA transfection. “4” is clone # 4 in which BRCA1 silencing was optimal 6 months post shRNA transfection. **C.** t-circles in cells after BRCA1 silencing. Genomic DNA was extracted from the different clones and was separated in 2D gel system. Subsequently, gels were blotted onto a positively charged membrane and blotted with a probe containing a complementary sequence to that of telomeric repeats. The upper left panel is a cartoon explaining the migration of t-circles in agarose gels. The arrows point to the linear double stranded DNA, the single stranded telomere and the telomeric circles (relaxed circle). WT”- the wild type intact cells; “NC”- cells transfected with the negative control shRNA plasmid; “KD” is clone # 4 in which BRCA1 silencing was optimal one month post shRNA transfection. **D.** t-circles formed by phi29 DNA polymerase. Genomic DNA was extracted from the different clones and was subjected to phi29 DNA polymerase which replicated telomere circles to concatameres that migrate as a very heavy band in an agarose gel. PC- a positive control from a telomerase negative cell line, U2OS which is rich in t-circles.

The cellular levels of γH2AX, a histone marker for DNA damage were measured both in the mononuclear cells of BRCA1/2 carriers and in HB-2 cells following BRCA1 silencing. In both experimental systems the levels of the phosphorylated histone γH2AX were markedly increased (140% in the patients relatively to the control group and in *in vitro* system the levels were elevated by 100% relatively to control cells). These results suggest an active ongoing DNA damage response process in non-malignant BRCA1/2 mutated cells (Figure 3A, 3B).

### T-circles are increased in BRCA1 silenced cells

T-circles results from trimming of too long telomeres. In addition, they characterize a non-stable state of telomeres paradoxally contributing to their elongation. To assess whether BRCA1 silencing is associated with the appearance of t-circles we subjected DNA isolated from the BRCA1 silenced cells to two dimensional gel electrophoresis. As shown in Figure 3C, t-circles appear in the BRCA1 silenced derived DNA and not in the controls. The number of t-circles was evaluated also by an additional method which verified the findings (Figure 3C). These results, together with the increase in γH2AX, imply DNA damage and dis-regulation of telomere homeostasis in these cells.

### The G-strand overhang is shorter in BRCA1/2 mutations carriers and in BRCA1 silenced cells

**Figure 4:**
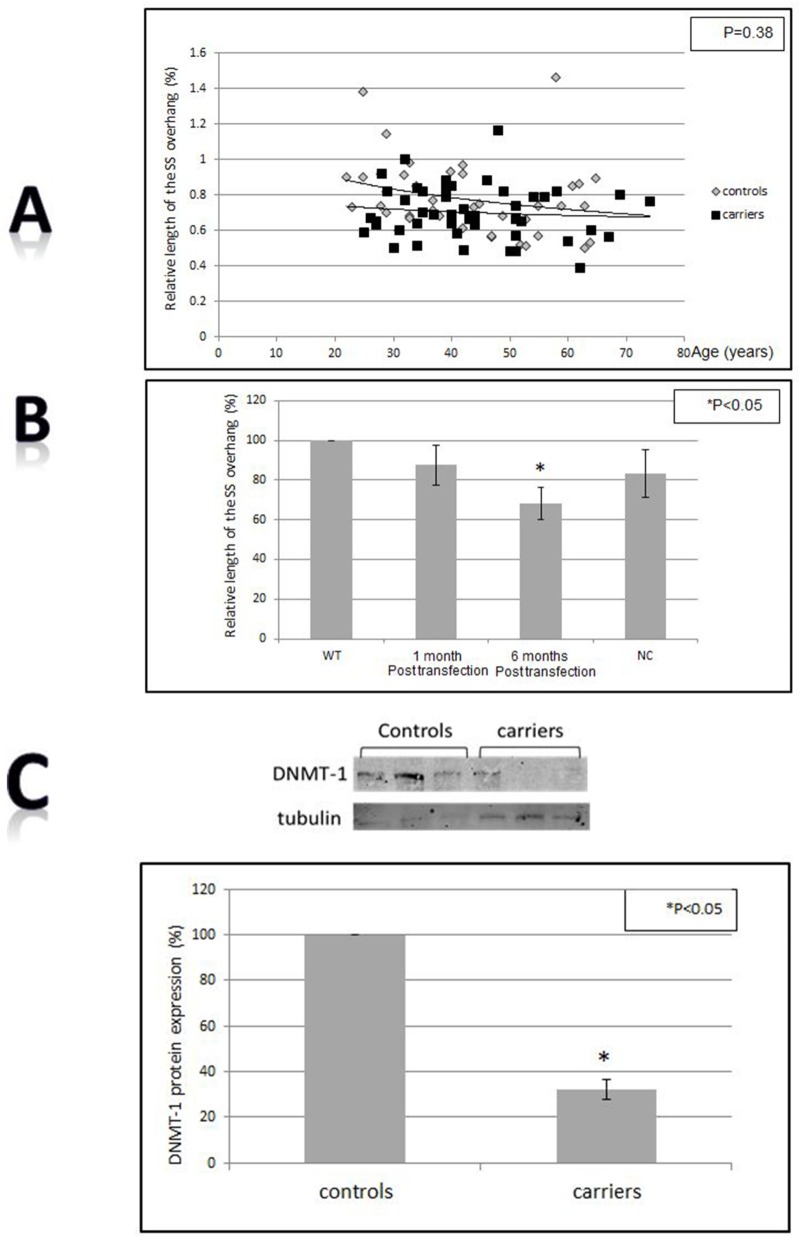

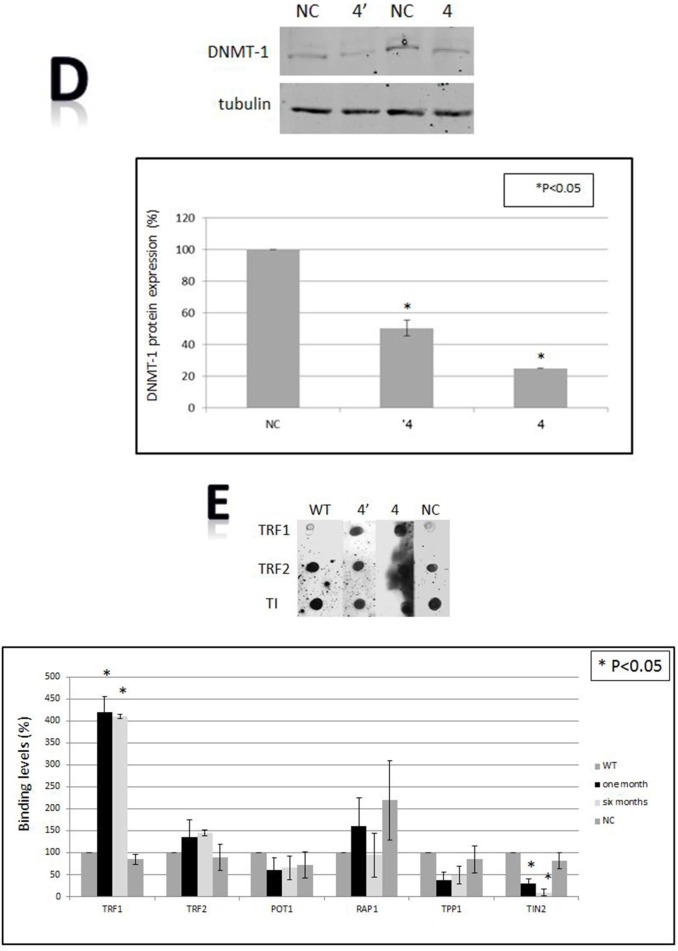
Telomere structure, sub-telomeric methylation and the shelterin proteins binding to telomeres **A.** Single stranded (SS) overhang length in BRCA1/2 carriers vs control. **B.** SS overhang after BRCA1 silencing in HB-2 breast epithelial cells. The length of the SS overhang was measured in both systems as described in the Method section by the luminescence obtained from the binding of a PNA probe to the DNA isolated from all samples and measured by luminometer. WT- the wild type intact HB-2 cells, NC- HB-3 cells transfected with a scrambled negative control plasmid. **C.** Levels of DNMT-1 in BRCA1/2 mutations carriers. The upper panel is an example of one Western blot and the lower is a quantitation of three independent experiments. Telomere structure, sub-telomeric methylation and the shelterin proteins binding to telomeres. **D.** Levels of DNMT-1 after BRCA1 silencing. The upper panel is an example of one Western blot and the lower is a quantitation of three independent experiments. “NC” depicts the cells which were transfected with the negative control shRNA plasmid; “4” “ is clone # 4 in which BRCA1 silencing was optimal one month post shRNA transfection. “4” is clone # 4 in which BRCA1 silencing was optimal 6 months post shRNA transfection. **E.** Binding of the shelterin members to telomeres. Cells were subjected to the ChIP assay analysis using specific shelterin proteins antibodies and a DIG- telomere complementary probe. The upper panel contains a representative ChIP-dot blot assay and the lower is a quantitation of three independent ChIP assays. “WT” - the wild type intact cells; “NC” - cells transfected with the negative control shRNA plasmid; “4” - clone # 4 in which BRCA1 silencing was optimal one month post shRNA transfection. “4”- clone # 4 in which BRCA1 silencing was optimal 6 months post shRNA transfection.

The length of the 3′ overhang at telomeres confers stability to the 3D structure of telomeres and is required for DNA protecting function of telomeres. The 3′ overhang was significantly shorter in the BRCA1 silenced cells (Figure 4B) (P<0.05 two-tailed T-test). In the BRCA1/2 mutations carriers group the single strand was also shortened but the difference was less pronounces and bordered on statistical significance (P=0.038 Levene’s test for equality of variants, Figure 4A, 4B). This finding implies damage to telomere structure imposed by the absence of active BRCA1 protein.

### The level of DNMT-1 is lower In BRCA1/2 mutations carriers and *in vitro*

DNA methylation is an important mechanism both in carcinogenesis and telomere length regulation. There were significant differences in the levels of DNMT-1between normal controls and BRCA1/2 mutations. As depicted in Figure 4C BRCA1/2 mutations carriers exhibited significantly lower levels of DNA methyl transferase-1 (DNMT-1) - about 45% of the control group. A similar change in DNMT-1 levels was observed after BRCA1 silencing (Figure 4D) in the *in vitro* system. These different levels may imply changes in methylation status that can affect telomere homeostasis and propensity to malignant transformation.

### The methylation status of the sub-telomeric region is different in BRCA1/2 mutations carriers and in silenced BRCA1 cells

The methylation status of the sub-telomeric region may play a role in regulation of telomere length. We assessed the methylation status of these regions in chromosomes 5 and 10 in both peripheral blood mononuclear cells of BRCA1 carriers and in BRCA1 silenced mammary epithelial cells. All carriers and controls were at the ages of 35-45 years at the time of blood sampling. The methylation pattern in the carriers suggested different levels compared to controls (Table 2). In carriers the subtelomeric region of chromosome 10 lost all methylation sites while the methylation status of chromosome 5 sub-telomeric region tended to be higher than that in the control group. In contrast, in BRCA1 silenced cells the methylation status in both chromosomes did not change.

**Table 1:** Patients’ characteristics

Parameter	BRCA1 carriers (n= 65)	BRCA2 carriers (n=27)
Age (mean ± SD)	**40** ± **12**	**51** ± **30**
185 del AG mutation	**47**	
5382 ins c mutation	**8**	
EX-18-20 mutation	**1**	
Unknown type of mutation	**4**	**1**
6174 del T mutation		**26**
Developed malignancy	**6**	**2**

**Table 2:** Methylation status of sub-telomeric region in BRCA1 carriers versus controls and in BRCA1 silenced cells versus controls

sample	Methylation of sub-telomeric region of chromosome 10 (%)	Methylation of sub-telomeric region of chromosome 5 (%)
Carrier	50.5	**0**
Carrier	100	**0**
Carrier	100	**0**
Carrier	100	**0**
control	100	17.1
control	47.4	14.9
control	75.4	18.3
control	84.3	11
WT cells	100	**0**
Negative control	118	**0**
One month post transfection	178	**0**
six month post transfection	120	**0**

### The shelterin members binding to telomeres after BRCA1 knockdown

We measured the binding to telomere of all six members of shelterin complex in HB-2 cells after BRCA1 silencing, as all of them possess a regulatory role in telomere maintenance. The level of binding to telomeres was measured by the Chromatin Immunoprecipitation (ChIP) assay (Figure 4E). Whereas the binding of four proteins (TPP1, POT1, TRF2 and PARP1) did not change significantly, TRF1 binding was markedly increased (four times relatively to control). In contrast, TIN 2 binding to telomere was reduced by 10 fold after BRCA1 silencing (Figure 4E). These finding may have bearing on telomere length regulation. The total cellular level of TRF1 and TIN 2 did not change as measured by Western blot (not shown).

### Gene expression profiling in cells following BRCA1 silencing

**Figure 5:**
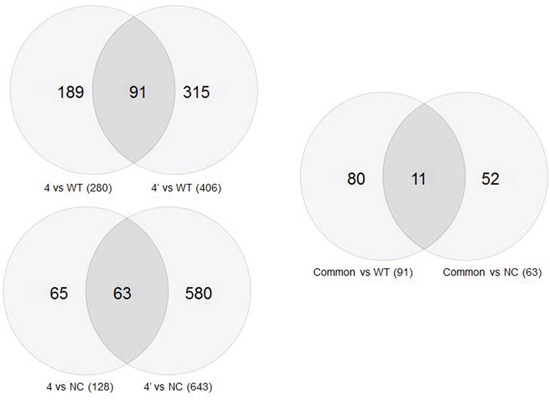
Venn circles representing gene expression of the various clones after BRCA1 silencing Comparisons of the number of genes whose expression is common to various samples in Venn diagrams. The upper left panel shows genes common to the wild type cells and cells in which the BRCA1 gene has been silenced, either for one month (4′) or for six months (4). The bottom Venn diagram symbolizes a similar comparison between gene expression in the negative control cells and the BRCA1 silenced cells. The right diagrams compares the upper and the lower diagrams.

Gene expression was analyzed in untreated cells, cells that were transfected with a negative control vector, cells in which BRCA1 was silenced a month and six months before gene expression analysis (Figure 5, Supplementary Figure S3). Figure 5 depicts the common genes between the various experimental groups. These common genes may imply on the dynamics of changes occurred throughout the six months of BRCA1 silencing in these cells. The expression of more genes was changed one month post BRCA1 silencing compared to the change after six months in comparison with either the intact wild type cells (91 genes) or the cells which were transfected with scrambled plasmid (63 genes). The third Venn diagram compares these differences.

The 11 genes which overlap between these two overlaps and their biological roles are listed in Table 3A. Eight genes that their expression was most significantly changed were successfully validated in our laboratory by qPCR. These include the following: Serpine (upregulated by 103%), DRB5 (downregulated by 40%), MLL3 (downregulated by 72%), TLE4 (downregulated by 28%), ANGPLT2 (upregulated by 184%), GPRC5B (downregulated by 15%), ABCA12 (downregulated by 63%) and BLNK (downregulated by 47%). 708 genes were differently expressed in BRCA1 silenced cells as compared with negative control. Inspection of their clustering at 1 and 6 months after silencing shows a gradual change in gene expression over time (Supplementary Figure S3A, S3B). Some of the genes that their expression was changed after 1 month returned to baseline levels after 6 months.

**Table 3A:** The biological function of differentially expressed genes among all samples

Gene	function	% change in BRCA1 silenced vs. controls
LPHN2	Latrophilin is a G-protein coupled receptors which may function in cell adhesion and signal transduction [77].	downregulated by 95% (P=4.69E^-09^)
MMP7	Matrix metallopeptidase 7 is involved in the breakdown of extracellular matrix in normal physiological processes as well as in disease processes such as arthritis and metastasis [77].	upregulated by 493% (P=1.20E^-05^)
HSD17B6	Hydroxysteroid (17-b) dehydrogenase 6 homolog has both oxidoreductase and epimerase activities and is involved in androgen catabolism [77].	upregulated by 400% (P=4.59E^-05^)
GPRC5B	G protein-coupled receptor may mediate the cellular effects of retinoic acid on the G protein signal transduction cascade [77].	upregulated by 789% (P=7.25E^-09^)
DHRS9	Dehydrogenase/reductase, may play a role in the biosynthesis of retinoic acid [77].	Downregulated by 73% (P=1.6E^-04^)
CCL20	Chemokine ligand 20 is a chemotactic factor that attracts lymphocytes and neutrophils. It Inhibits proliferation of myeloid progenitors. May be involved in formation and function of the mucosal lymphoid tissues by attracting lymphocytes and dendritic cells towards epithelial cells [77].	Downregulated by 80% (P=1.99E^-05^)
ABCA12	ATP-binding cassette, sub-family A is a membrane-associated protein, a member of the superfamily of ATP-binding cassette transporters and is involved in transporting various molecules across extra- and intracellular membranes [77].	downregulated by 500% (P=9.25E^-08^)
HERC5	A E3 ubiquitin protein ligase 5 which is upregulated in endothelial cells by a pro-inflammatory cytokine. Functions as an interferon-induced E3 protein ligase that mediates ISGylation of protein targets. It acts as a positive regulator of innate antiviral response in cells induced by interferon [77].	upregulated by 350% (P=2.02E^-06^)
SLC7A11	Solute carrier family 7, a member of cysteine and glutamate anionic transport system. The predominant mediator of Kaposi sarcoma-associated herpesvirus fusion and entry permissiveness into cells. Increased expression of this gene has been shown in primary glioma [77].	downregulated by 68% (P=2.8E^-04^)
GDA	Guanine deaminase is responsible for the hydrolytic deamination of guanine and may play a role in microtubule assembly [77].	Upregulated by 931% (P=1.50E^-08^)
LRCH2	A member of the leucine-rich repeat and calponin homology domain-containing protein family which contains multiple N-terminal leucine-rich repeats, in addition to a C-terminal calponin homology domain, a type of domain that mediates interactions with actin filaments, functioning as a cytoskeletal scaffold [77].	upregulated by 821% (P=1.08E^-11^)

A heat map of DNA repair related genes [obtained from ref 30] reveals a distinct pattern in the silenced cells implying a certain dynamics related to DNA repair pathways upon silencing of BRCA1 gene (Supplementary Figure S3C).

Analysis of the functions of the differently expressed genes in our samples showed that genes related to transcription or nucleic acids processes were overexpressed after one month of BRCA1 silencing. These genes were mostly downregulated six months after BRCA1 silencing and other genes, related to nucleotide binding and response to stress were the most upregulated six months of BRCA1 silencing (Supplementary Figure S3D)

## DISCUSSION

Our study demonstrates that BRCA1/2 mutations cause wide array of perturbations in telomere structure and dynamics. These perturbations are associated with constitutive DNA damage response in nonmalignant cells of BRCA1/2 mutations carriers without cancer as well as in *in vitro* system. We did not find difference between BRCA1 and BRCA2 mutation carriers and the results were similar also in BRCA1 silenced cells. Therefore we assume that the effects on telomere biology are common to BRCA dysfunction *per se*.

The *in vitro* data prove that BRCA dysfunction abolishes telomere length homeostasis. In contrast to normal cell cultures in which telomere length is stable due to active telomerase coupled with homeostatic mechanisms, in BRCA1 silenced cells telomere length increased gradually, reflecting loss of telomere length homeostasis. Since telomerase is inactive in human somatic cells, the telomere length in BRCA1/2 mutations carriers did not increase, but the physiological telomere shortening was abolished.

The literature on telomere length in BRCA1/2 mutations carriers is conflicting. Pooley et al. found a tendency to longer telomeres in BRCA1/2 mutations carriers compared with the normal population [31]. Blasco and coworkers [32] reported on shortened telomeres and anticipation in BRCA1/2 mutations carriers [33]. Another study did not find differences in telomere length between BRCA1/2 mutation carriers and normal population [34]. However, none of these studies dealt with age related dynamics of the subjects. In this respect the results of our study are novel describing the abnormalities in time related telomere dynamics. The *in vitro* results of our study are in keeping with others showing telomere elongation in BRCA1/2 mutations or knockdown in several cell types [26, 35, 36, 27] thus confirming the *in vivo* results.

Abnormalities in telomere dynamics were also reflected by the length distribution pattern of telomeres within different chromosomes. As seen in Figure 1C this distribution is more heterogeneous in BRCA1/2 mutation carriers. Greater heterogeneity of telomere lengths is also apparent in the 2D gels (Figure 3C). The degree of heterogeneity of telomere length within cells has been reported to be associated with breakage-fusion-bridge cycles and telomeric fusions, phenomena important in the process of malignant transformation [37, 38]. In addition, BRCA1 mutation was associated with more telomere free endings (Figure 1D) representing very short telomeres which are usually associated with telomere dysfunction and DNA damage [39]. Another perturbation that may lead to telomere dysfunction was the finding of shortened single stranded overhang both in BRCA1/2 mutations carriers and the BRCA1 silenced cells. Indeed, both in *in vivo* and *in vitro* cells, BRCA1/2 mutation were associated with increased DNA damage as evidenced by elevated levels of H2AX protein, in keeping with previous reports [35, 11, 27]. All the before mentioned telomere perturbations probably contribute to this constitutive DNA damage.

Several mechanisms may contribute to the homeostatic defect of telomere length in BRCA1/2 mutation. Telomerase activity is a major determinant of telomere length [40]. Previous studies reported that overexpression of BRCA1 inhibited telomerase activity by transcriptional mechanism [27]. Conversely, decreased BRCA1 expression increased telomerase expression in telomerase positive mammary epithelial cells and telomerase negative cells [35]. We did not find increased telomerase activity in BRCA1 silenced cells or different inducibility of telomerase activity in BRCA1/2 mutations carriers compared to normal population suggesting that BRCA1/2 mutations do not significantly affect telomerase activity or regulation.

Other important regulators of telomere length are the shelterin proteins [16]. ChIP assay demonstrated decreased binding of TIN2 and increased binding of TRF1 to telomeres in BRCA1 silenced cells. The total amount of TRF1 in the cell did not increase suggesting that the increased binding to telomere is a specific finding. The decrease in TIN2 binding to telomeres is in keeping with their elongation in the cell culture. TIN2 binds to telomeres indirectly via TRF-1 and acts as a telomere length regulator. Campisi et al. [41] have shown that a mutant TIN2 cannot properly regulate telomere length and therefore cells harboring this mutant possess longer telomeres. We suggest that a similar phenomenon occurs in our setting: decreased TIN2 binding to telomeres affects telomere length homeostasis by promoting their elongation in telomerase positive cells (such as HB-2). TRF1 is a negative regulator of telomere length [42] but in our setting this function was probably masked by the activity of TIN2. The mechanisms of altered TIN2 and TRF1 binding to telomeres are beyond the scope of this study and should be addressed in the future.

An increased number of t-circles found in BRCA1 KD cells may be related to telomere elongation and telomere dysfunction. Reddel *et al*. [43] showed that abnormal telomere elongation leads to increased telomere trimming manifested by formation of t-circles. T-circles may be related to telomere elongation also in additional mechanism. The replication of t-circles by roll- and- spread mechanism represents another, less studied, way of generation of long telomeres [44]. T-circles have been shown to promote telomere elongation in vivo in *K. Lactis* [45]. These circles have been shown to be associated with high level of telomere instability in mutants of *K. Lactis* lacking stn-1 protein [44]. Human t-circles have been propose to serve as an indicator for rapid telomere deletion (RTD) by telomere trimming, another regulatory mechanism for telomere length regulation characterizes mainly ALT (Alternative Lengthening of Telomeres) cells [46]. BRCA1/2 dysfunction may affect the formation of t-circles by RTD, circles that serve as template for telomere elongation by the rolling circle mechanism. This exact mechanism was described in the *K. Lactis stn-1* mutants [44].

Methylation of subtelomeric regions was also reported to play a role in telomere length regulation [22, 47] and in malignant transformation [48]. DNMT-1 levels were significantly lower both in the carriers' mononuclear cells and in the silenced mammary epithelial cells. This decrease may be one of the causes of perturbed telomere length homeostasis as in DNMT-1-deficient cells demethylation of subtelomeric regions induced telomere elongation [22]. There were also changes in methylation of the subtelomeric regions, albeit not clear cut and these changes were manifested only in the carriers and not in the *in vitro* system.

The subtelomeric region of chromosome 10 lost all of its methylated sites in BRCA1/2 mutations carriers. This difference was not due to age related process since the patients and the controls were in the same age group. The methylation pattern in chromosome 5 suggested increase in methylation but the results were not consistent and due to the small number of samples we cannot reach any definite conclusion. However, data from the literature support the finding of methylation dynamics caused by BRCA mutation. Anjum *et al*. found that a specific DNA methylation signature in lymphocytes is associated with poor prognosis of BRCA1 mutation carriers [49]. In addition, a specific signature of methylated genes that predict breast cancer risk was recently identified in BRCA1 mutation carriers in two separate studies [49, 21]. The changes in the methylation of the promoters of the selected genes varied: some exhibited increase and others a decrease in their methylation status, similar to our variable results.

The results of this study demonstrate that BRCA mutations affect the biology of DNA methylation at least in the subtelomeric regions. These perturbations are probably associated with the process of malignant transformation. The relation between changes in DNMT-1 levels and the methylation status shown in our study is yet unclear, but consistent with other reports [50]. A similar phenomenon was demonstrated in mice where BRCA1 was shown to bind to the promoter of the DNMT-1 gene through a potential OCT1 site [51].

Ours are the first published data of gene expression in BRCA1 silenced nonmalignant breast epithelial cells. It is of interest to compare these results to those obtained from BRCA1 mutation carriers. Knudson et al. [52] published gene expression analysis from breast specimens of six BRCA1 mutations carriers. Among the top 10 genes that were differentially expressed in the carriers five were also differentially expressed in our analysis. These genes include the CHI3L1 (Chitinase 3-like 1), MUC1 (Mucin 1), KLK1 (Kallikrein-related peptidase 1), ANXA8 (Annexin A8) and MUC16 (Mucin 16, cell surface associated). Functions of some of these genes may play a role in the process of malignant transformation (Table 3B).

**Table 3B:** The biological function of differentially expressed genes in the BRCA1 silenced cells and *in vivo* in BRCA carriers [ref 32]

Gene	function	% change between samples
CHI3L1	Chitinase 3-like 1 has a proliferative role in stromal fibroblasts and chemotactic effects on endothelial cells. It is involved in angiogenesis and was found to be upregulated in the sera of patients with glioblastoma [77].	upregulated by 107% (P=8E^-03^)
MUC21	Mucin 21 was reported to be over-expressed in esophageal squamous epithelia and carcinomas [78].	downregulated by 75% (P=1.69E^-06^)
KLK1	Kallikrein-related peptidase 10 KLK1, a serine protease which is implicated in carcinogenesis and may potentially serve as a novel cancer and other disease biomarkers [77].	upregulated by 113% (P=4.5E^-02^)
ANXA8	Annexin A8 may function as an anticoagulant that indirectly inhibits the thromboplastin-specific complex. Overexpression of this gene has been associated with acute myelocytic leukemia [77].	downregulated by 44% (P=1.5E^-04^)
MUC15	Mucin 15 cell-to-matrix adhesion, is associated with poor prognosis of glioma [79]	downregulated by 82% (P=7.38E^-06^)
TGFβ	plays an important role in carcinogenesis in general and in breast cancer progression in particular [80]	upregulated by 270% (P=1.65E^-06^)

Changes are between the clones in which BRCA1 was silenced for either one or six months versus the WT intact cells.

In addition to those 5 genes found both in the Knudsen and our studies, gene expression profiling in our cells yielded several genes whose expression was significantly altered. Among those is TGFb which is known to play an important role in carcinogensis in general and in breast cancer progression in particular [53–55]. Other genes whose expression was significantly altered may have a biological relevance to cancer and metastasis. MMP7 codes for a metastatic promoting protein, HERC5 is an ubiquitin ligase that may be involved in malignancy, SLC7A11 is a part of the anionic amino acid transport system and was highly expressed in early stages of glioma, and LPHN2 has a role in cell adhesion. The possible involvement of these genes in the effects of BRCA1 silencing on telomere dynamics is still unknown but will be addressed in future studies in our laboratory.

We did not observe gene expression variations that may add to our understanding of the perturbations in telomere structure and dynamics described in this study.

Are these perturbations described in this study connected to the propensity of BRCA1/2 mutated cells to undergo malignant transformation? The shortening of single stranded overhang, increased number of free telomere ends and increased length heterogeneity may all contribute to tumor promoting processes in these cells.

Telomere lengthening or lack of shortening does not seem to play a role in carcinogenesis. Other reports showing that in breast cancer cells the telomeres are not shortened as they are in almost all other cancers also suggests that the loss of telomere length homeostasis is indeed a result of BRCA mutation but probably does not contribute to the process of malignant transformation. Strengthening this notion is our finding that in BRCA1/2 mutations carriers who eventually developed breast cancer we did not find additional changes in telomere length in mononuclear cells (not shown). The shortening of the single stranded overhang, however, may cause telomere dysfunction and initiate DNA damage. This process is known to be associated with carcinogenesis [56–58]. The evidence of increased degree of DNA damage both in carriers and in the *in vitro* cells lends support to the possible role of single stranded overhang shortening in the carcinogenesis of BRCA1/2 mutation carriers.

The changes in DNMT-1 levels and associated change in methylation may also contribute to carcinogenesis. Lending credibility to this assumption is the fact that hypermethylation of the gene promoter is a well-known epigenetic event and is a critical regulatory component in pathological processes such as cancer mediating inactivation of tumor suppressor genes or activation of growth-promoting oncogenes thus promoting tumorigenesis [59, 60].

We do not know whether the changes in binding of two shelterin proteins, TRF1 and TIN2 to telomeres have relevance to tendency to malignant transformation of the BRCA1/2 mutated cells. A possible connection may be the association between TIN2 and mitochondrial function. TIN2 is posttranslationally processed in the mitochondria and regulates mitochondrial oxidative phosphorylation [61]. The importance of mitochondrial metabolism in cancer has been recently highlighted in the literature [62]. TRF1 was shown to be required for overexpressed Nek2 to trigger abnormal mitosis and chromosomal instability thus promoting carcinogenesis [63]. In colorectal cancer cells isolated from surgical specimens, the levels of TRF1 were higher compared to those of normal tissues while average telomere length was lower in these cells [64]. Finally, changes in telomere binding proteins including TRF1 and TIN2 which correlated with telomere lengths were observed in breast carcinomas [65, 66].

A possible implication of the various genes that their expression was changed after BRCA1 silencing is depicted on Table 3A, 3B which describes the biological function of a selected number of genes such as cell adhesion, enhanced proliferation and more.

**Figure 6:**
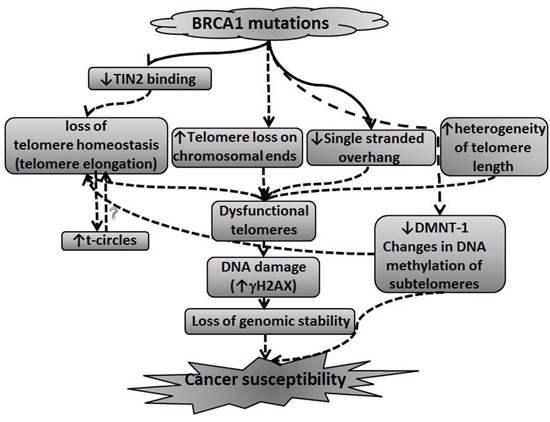
A model connecting BRCA1/2 mutations, telomere perturbations and malignant transformation This model summarizes the possible connection between BRCA1/2 mutation leading to perturbations in telomere length homeostasis and susceptibility to malignant transformation. The model is described in more details in the Discussion section.

A model summarizing the possible connection between telomere perturbations caused by BRCA mutation and carcinogenesis is depicted in Figure 6. According to this model BRCA1/2 mutation causes several changes leading to perturbation in telomere length homeostasis. These are 1) a decrease in DNMT-1 levels and subsequently changes in methylation of subtelomeric regions, 2) Decrease of TIN2 binding to telomeres, 3) increase in telomere trimming manifesting as t-circles also contributes to loss of telomere homeostasis. In addition, BRCA1/2 mutation causes shortening of single stranded overhang, heterogeneity of telomere lengths and increased telomere loss on chromosomal ends. These perturbations and the loss of telomere homeostasis cause telomeres to be dysfunctional resulting in persistent DNA damage in the cells. The DNA damage state and ensuing loss of genomic stability with changes in methylation contribute to susceptibility to malignant transformation.

In summary, we found that BRCA1/2 mutations cause significant perturbations in telomere biology. These perturbations or their biological correlates may underlie, at least to some extent the process of development of cancer in those cells and carriers.

## MATERIALS AND METHODS

### BRCA1/2 mutations carriers

84 healthy female carriers of the BRCA1/2 mutation attending our BRCA clinic and an age matched control group of 64 healthy women were recruited to the study. The study was approved by the Institutional Review Board. Patients' characteristics are shown in Table 1.

### Processing of blood samples

The mononuclear cells were isolated from 15 ml of blood using the Ficoll-Hypaque gradient method [30]. DNA, protein and whole cells were kept for further analyses.

### Telomerase activity

The activity of telomerase was calculated by a real-time PCR based TRAP assay (QTD Kit; Allied Biotech, Taipei, Taiwan). For calculation of telomerase activity TSR standard curve was prepared using 1:5 serial dilutions of the stock concentration with lysis buffer, (0.5-0.00016 amoles/μl). The PCR program included: 20’ at 25°C, 10 minutes at 95°C and then 40 cycles each lasts for 30’’ of 95°C, 60°C and 72°C. Telomerase activity was calculated using TSR standard curve equation and C_t_ values of the samples.

### Exposure of mononuclear cells to PHA

Whole blood was exposed to PHA (Biological Industries, Beit Haemek, Israel) for 48h to induce telomerase activity [67]. Cells were harvested and subjected to TRAP assay. Nine BRCA1/2 mutations carriers and ten age matched controls were chosen for this assay.

### Q-FISH assay for telomere length

Q-FISH was performed according to the method of Lansdorp et al. [68] with modifications. Basically, metaphase spreads were prepared after growing the cells in the presence of PHA for three days and hybridized to a TTAGGG PNA probe labeled with Cy-3 (Panagene, Inc. Daegeon, Korea). The slides were stained with DAPI and visualized under the fluorescent microscope. Quantitation was done on 10 fields of each patient’s sample with the TFL-telo2-2[1] software [68]. Samples from 10 BRCA1 mutation carriers were compared with 9 age matched controls. The results of the software analysis were further manually verified and corrected in cases of ambiguous telomere signs.

### Telomere length by flow-FISH

Measurement of telomere length was performed by Telomere PNA Kit/FITC for flow cytometry (Dako, Glostrup, Denmark) according to the manufacturer’s instructions. Samples were analyzed using the FACSCALIBUR (Becton Dickinson, San Jose, CA, USA) device.

### The single stranded overhang length

The single stranded telomere overhang length was measured according to the G-telomere HPA method developed by Tahara and colleagues [69] with slight modifications. Basically, 7.5 mg DNA was suspended in TE and sheared with a 25G syringe. 20 fentomoles of acridinium ester labeled (CCCTAA)_4_ probe equivalent to 3X10^5^ RLU was used for each hybridization reaction. Hybridization took place in 60°C water bath with no agitation for 20 min. Hydrolysis buffer was then used to wash unbound probe and samples were incubated at 60°C in a water bath with no agitation for 10 min. Samples were divided into three aliquots and loaded onto 96 wells black plate. Reagents from the Gen-Probe detection kit (Gen-Probe Incorporation, San Diego, USA) were automatically loaded to each well and total luminescence was read by using the Microwin 2000 software in Berthold Centro XS LB 960 luminometer.

### Western blotting

Cells were lysed using protein lysis buffer and protein content was measured by the Bradford assay. 100g of anti-γH2AX antibody or anti-DNMT-1 (1:1000, Cell-signaling Inc., Danvers, MA, USA) and anti-GAPDH (1:5000, Santa Cruz Biotechnology Inc., Dallas, Texas) as a reference gene followed by hybridization with fluorescent labeled secondary antibody (LI-COR, Lincoln, NE, USA). Protein levels were then visualized and quantified (Odyssey IR imaging system; LI-COR, Lincoln, NE, USA). In addition to the BRCA1 silenced cells, several representative samples from BRCA1/2 mutations carriers and control groups were chosen for this assay.

**Figure:**
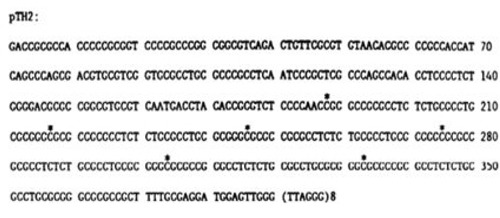


### Methylation of the sub-telomere region

The methylation of the sub-telomeric regions at chromosomes 5, 10 was analyzed using pyrosequencing (Nucleix LTD; Tel-Aviv, Israel). We used specific primers to amplify sub-telomeric regions containing multiple repeats of the following pTH2 sequence [70]. 9 age representative samples from BRCA1/2 mutations carriers and control groups were chosen for this assay.

### Cell cultures

HB-2 cells (breast epithelial nonmalignant cells, kindly provided by Prof. Adit Ben Baruch, Tel Aviv University, Israel) were grown with DMEM, supplemented with 20% fetal bovine serum (FBS), 1% penicillin/streptomycin and 1% glutamine, hydrocortisone, 2.6% insulin. HCC1937 cells harboring the BRCA1 mutation (kindly provided by Dr Tamar Rubinek, Sourasky Medical Center, Tel Aviv) were grown similarly. Cells were incubated in the presence of 5% CO_2_, 95% humidity at 37°C.

### BRCA1 silencing

To mimic the BRCA1 mutation status HB-2 cells were stably transfected with four different sh-RNAs against BRCA1 containing constructs and a scrambled sh-RNA containing plasmid as a negative control (Qiagen, Hamburg, Germany) by using Lipofectamine 2000^C^ (Life Technologies Inc., Grand Island, NY, USA). Transfection conditions were calibrated with GFP construct containing plasmid. Selection of transfected cells was done in the presence of 50 ng/ml puromicin (Sigma, Rehovot, Israel) in which 90% of the non-transfected cells are killed. BRCA1 protein level was measured by Western blot in order to choose the best BRCA1 silencing construct. Clone #4 exhibited the highest silencing efficiency (~30%) and was therefore chosen for our experimental system (Supplementary Figure S1). Cells were grown in the presence of 50 ng/ml puromicin. The silencing of BRCA1 was monitored periodically throughout the experiment and found to be stable (not shown). In addition, the proliferation rate, viability and the morphology of the transfected cells revealed no change throughout the whole experimental period compared with the parent clone (not shown).

### TRF measurement

Terminal Restriction Fragment (TRF) representing telomere length was measured by Southern blot with a DIG-labeled probe according to the manual provided in the TeloTAGGG Telomere Length Assay (Roche, Mannheim, Germany). Genomic DNA was extracted using the ArchivePure 5′ DNA blood kit (Hilden Deutschland) and quantified by the NanoDrop (Thermo, Waltham, MA, USA). 5 mg DNA was digested for 16h with *RSA*I and *HINF*I. The digested DNA was separated by gel electrophoresis (0.8% Agarose), depurinated by 0.25M HCl, denatured by alkaline denaturing solution (0.5M NaOH; 1.5M NaCl) and then neutralized in 0.5M Tris.HCl, 3M NaCL. Subsequently the DNA was capillary transferred onto a positively charged Whatman nylon membrane (Roche, Manheim, Germany) for 16hr and then UV-cross-linked (120mJ) to the membrane and incubated for 16hr with DIG-labeled TL probe (CCCTAA)_4_. Subsequently membrane underwent washes while agitated: twice in stringent wash buffer I (2X SSC, 0.1% SDS) for 5 min at RT, twice in stringent wash buffer II (0.2X SSC, 0.1% SDS) for 15 min at 50°C, in 1X maleic acid buffer for 5 min in blocking solution for 30 min at RT, in Anti-DIG-AP solution for 30 min at RT, twice in washing buffer for 15 min at RT, and finally in detection solution for 5 min at RT. The membrane was then applied with ~40 drops of CSPD substrate and exposed to a sensitive film for 1.5hr. The film was developed and then scanned and quantified by the Quantity One software (Versadoc; BioRad).

To calculate TRF each smear representing heterogenic signal was segmented and its intensity was measured. TRF was calculated according to the following equation:

$$\frac{\sum \left(O_{D_{i}}\right)}{\sum \left[\left(\frac{OD_{i}}{L_{i}}\right)\right]}$$

Where OD_i_ is the chemiluminiscent signal and L_i_ is the length of the TRF at position i.

### ChIP assay

The binding of the shelterin proteins to telomeres was measured by the ChIP assay (ChIP assay kit, EZ-ChIP; Millipore, Darmstadt, Germany). Briefly, the DNA was cross-linked to its bound proteins using formaldehyde. The cells were then collected, lysed and the DNA was sonicated to shear it to a size of 200-1000bp. The immunoselection was made using primary antibodies for each of the 6 shelterin proteins: 1μg of anti-TRF1, 1μg of anti-TRF2, 1μg of anti-RAP1, 1μg of anti-POT1 (all from Santa Cruz, Atlanta, GA, USA), 2μg of anti-TPP1 (Abcam, Cambridge, UK) and 4μg of anti-TIN2 (Proteintech, Chicago, IL, USA). Subsequently the complexes DNA-proteins-antibodies were precipitated by binding to protein-A-agarose beads. The DNA was then reverse cross-linked by incubation at 65°C. RNA and protein were degraded by RNAse and proteinase K respectively and the DNA was purified (QIAquick PCR Purification, Qiagen, Hamburg Germany). Eluted DNA samples were dot-blotted on positively charged Nylon membrane (Roche, Mannerheim, Germany). Subsequent steps of the assay were similar to those of the Southern blotting and included hybridization to the DIG-labeled telomeric probe and the detection of the chemiluminiscent signals.

### T-circles assay

The identification of t-circles was performed by two-dimensional agarose gel electrophoresis hybridization as previously described [71]. Basically, 20μg DNA was digested for 16h with *RSA*I and *HINF*I. The digested DNA was separated by gel electrophoresis (0.4% Agarose) for 12-16 hrs in the dark at room temperature. Lanes were cut out and separated in a second dimension in a 1.2% agarose gel for 6 hrs in the dark at 4°C. The DNA was transferred onto a nylon membrane for Southern blot assay as described above using a radioactive labelled telomeric probe. Signals were visualized both by phosphor imager (Fuji FLA-5000 system, Tokyo, Japan) and by film exposure.

In addition, we performed the ϕ29 DNA polymerase assay identified t-circles as previously described [46]. In principle, this assay allows an amplification of the t-circles by ϕ29 DNA polymerase and upon PAGE the circles form concatamers that migrate as a discrete band.

### Microarray hybridization and data analysis

The gene expression profiling was performed at the Genome Center at the Sackler School of Medicine and microarray analysis at the Bioinformatic Unit, The George S. Wise Faculty of Life Sciences, both in Tel Aviv University. Control untreated cells, cells that were transfected with scrambled containing plasmid, cells that were transfected for a month and six months with shRNA were subjected to the microarrays analyses. RNA was extracted in triplicates with the RNeasy mini kit (Qiagen, Hamburg, Germany) and measured by the Nanodrop (Thermo, Waltham, MA, USA), visualized on an agarose gel, aliquoted and hybridized to DNA microarrays (Affymetrix GeneChip® Human Gene 1.0 ST arrays) as described in the Affymetrix website (http://www.affymetrix.com). We used a total of 8 chips in triplicates. Microarray analysis was performed on CEL files using Partek® Genomics Suite TM, version 6.5 Copyright © 2010 (http://www.partek.com). Data were normalized and summarized with the robust multi-average method [72] followed by ANOVA. Cluster analysis of the array was obtained by Partek® Genomics Suite TM. Gene expression data were sorted using cutoffs of *p* < 0.05 under FDR (false discovery rate) adjustment criteria of *p* < 0.0002 for NDF and of *p* < 0.0005 for DF [73] and fold-difference cutoff of 1.5. We used Toppgene [74] for analyses of biological and functional groups. Gene ontology data were verified by Gather (Solinger JA) and by David [75] tools. Venny was used to cross between genelists [76]. All data is MIAME compliant and the raw data has been deposited in a MIAME compliant database: https://www.ncbi.nlm.nih.gov/geo/query/acc.cgi?acc=GSE26458

## SUPPLEMENTARY FIGURES AND TABLES


