# Supplementary material for: BRCA1/2 mutations perturb telomere biology: characterization of structural and functional abnormalities in vitro and in vivo
Source: Oncotarget. 2015 Oct 16;7(3):2433–54. doi: 10.18632/oncotarget.5693 (PMC4823046; doi:10.18632/oncotarget.5693)
Supplement: Supplementary file 1 [file oncotarget-07-2433-s001.pdf]

SUPPLEMENTARY FIGURES AND TABLE

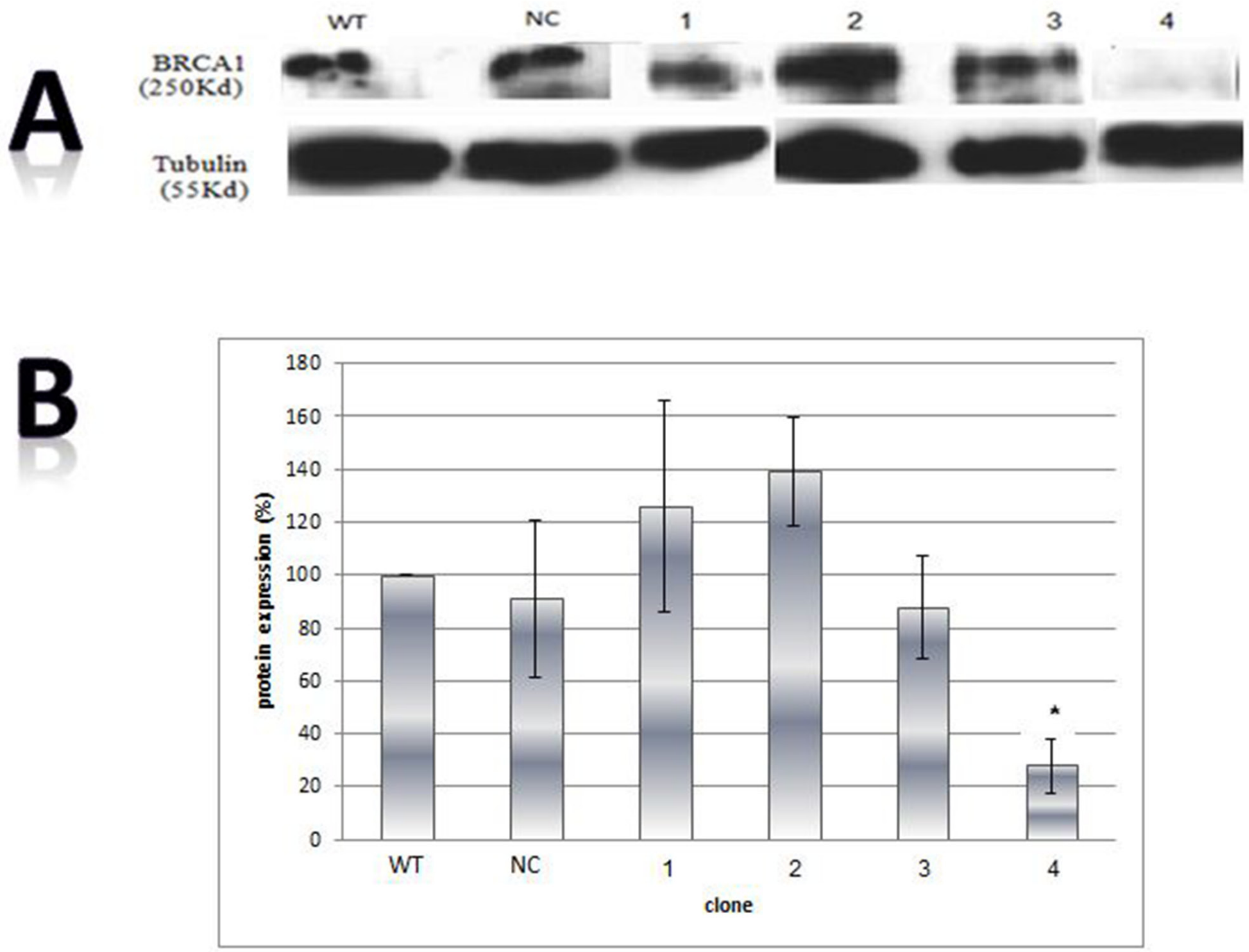

Supplementary Figure S1: BRCA1 silencing. A. An example of western blot representing BRCA1 expression of the various clones. **B.** Quantitation of A (two biological repetitions). "WT"- the wild type intact cells; "NC" depicts the cells which were transfected with the negative control ShRNA plasmid.

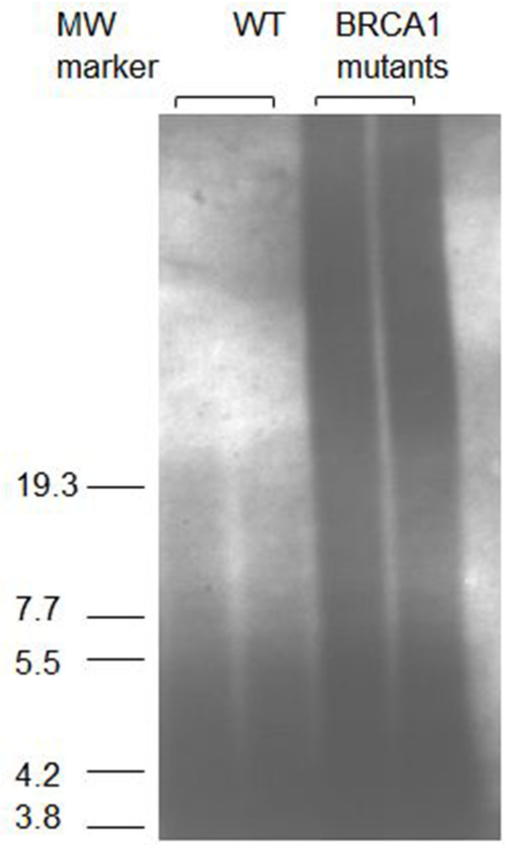

Supplementary Figure S2: Telomere length in cell line with BRCA1 mutation. Telomere length were measured in the wild type HB-2 cells as well as in the BRCA1 mutated cell lines by Southern blotting.

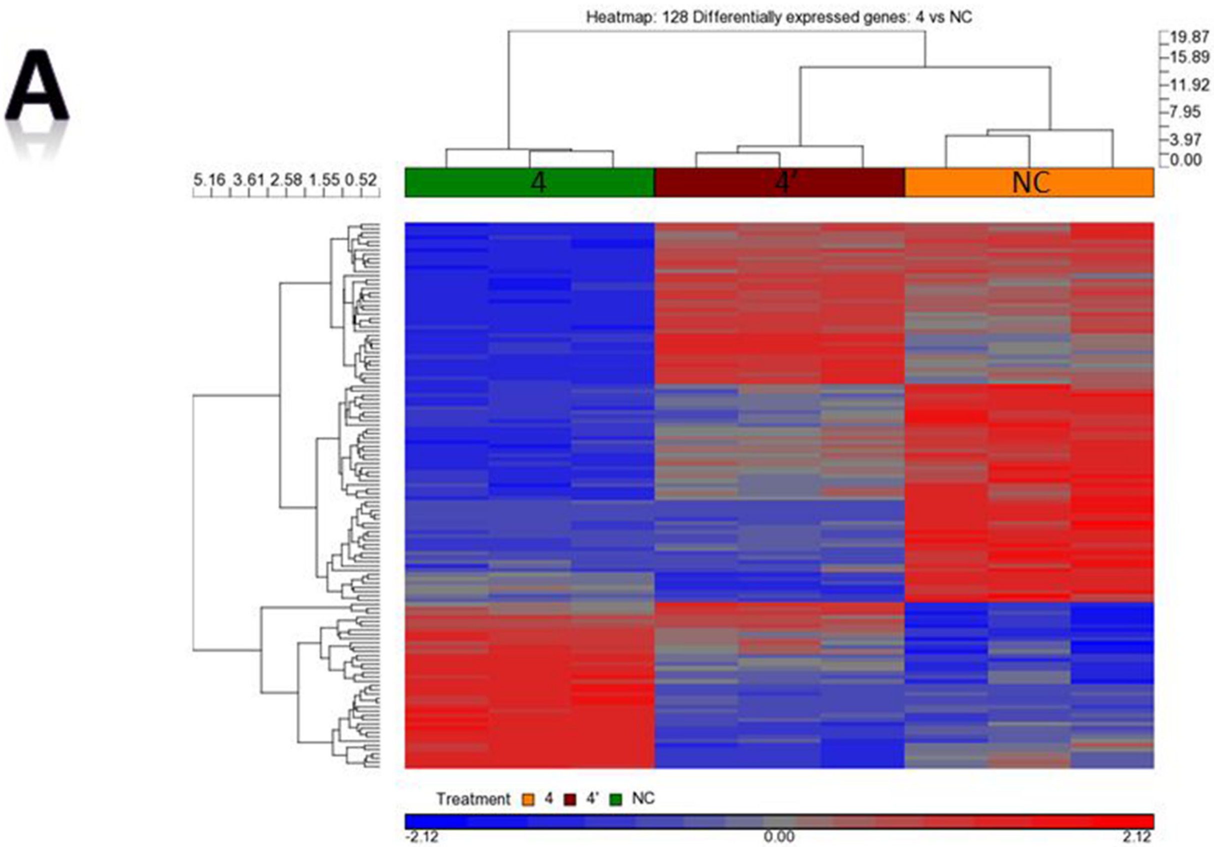
A
Heatmap: 128 Differentially expressed genes: 4 vs NC
19.87
15.89
11.92
7.95
3.97
0.00
5.16 3.61 2.58 1.55 0.52
4
4'
NC
Treatment 4 4' NC
-2.12
0.00
2.12

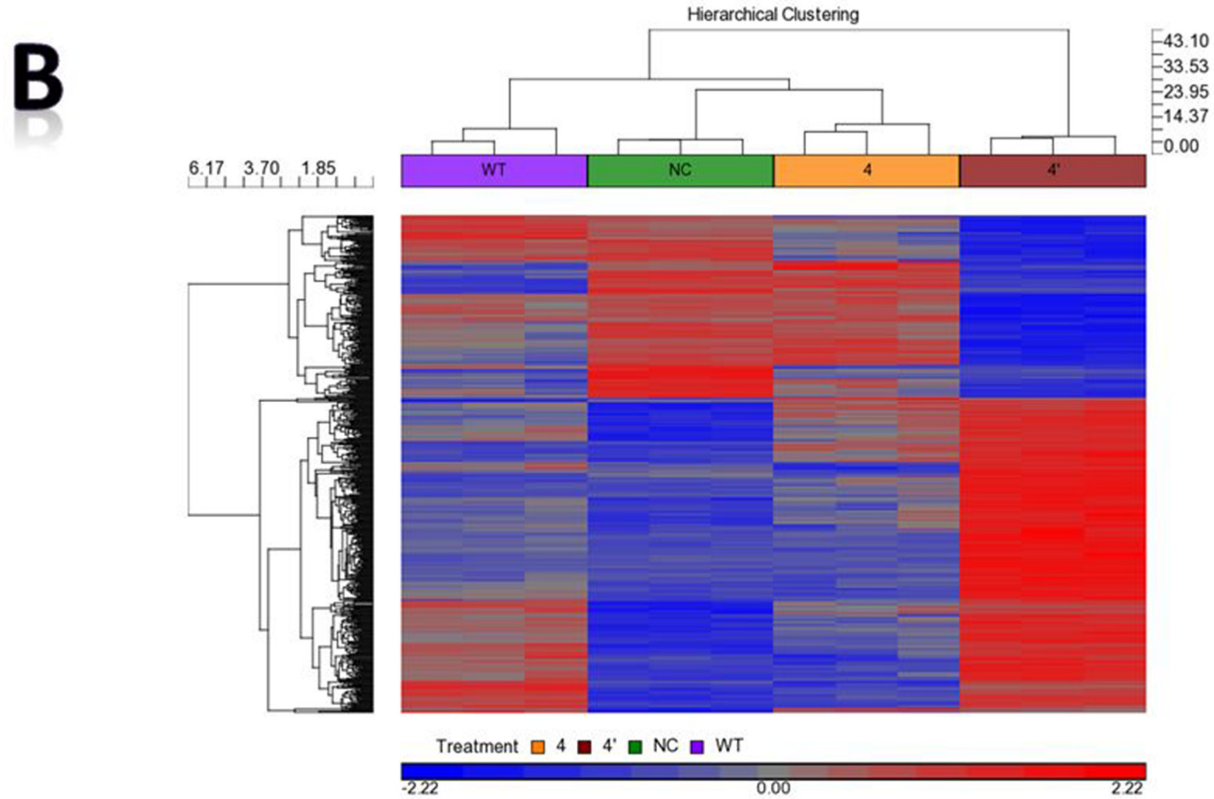
B
Hierarchical Clustering
43.10
33.53
23.95
14.37
0.00
6.17 3.70 1.85
WT
NC
4
4'
Treatment 4 4' NC WT
-2.22
0.00
2.22

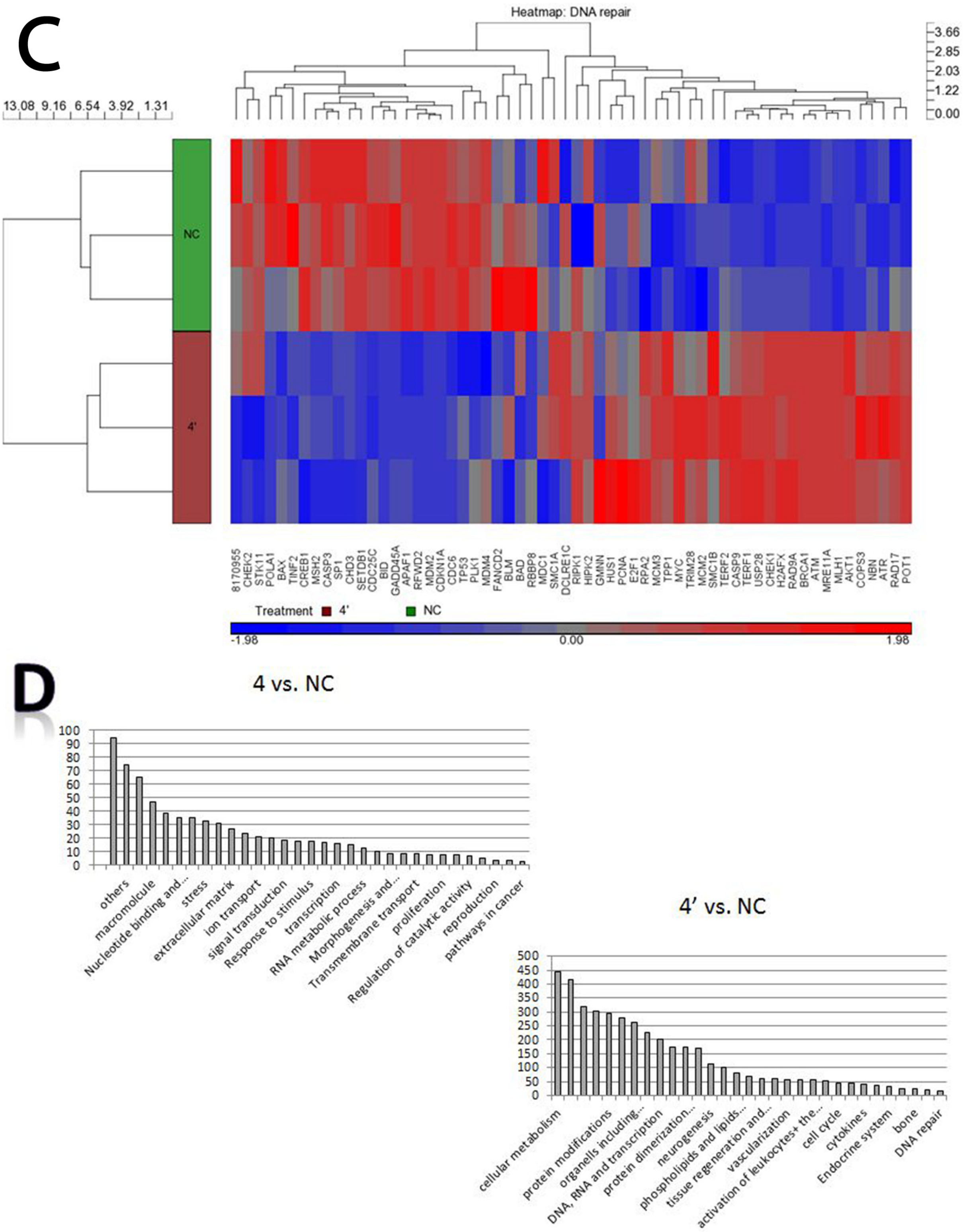

Supplementary Figure S3: Concrete signature of expressed genes in cells following BRCA1 silencing. A. Heat map of 128 differentially expressed genes: 4 versus NC; **B.** Heat map of 643 Differentially expressed genes: 4′, 4, WT, NC; **C.** Heat map of DNA repair related genes in 4′, NC; **D.** Gene functions in BRCA1 silenced cells and intact controls; **E.** Ven representation of all genes obtained with minimal cutoff of <0.05 and fold-change cutoff of 2. “WT”- the wild type intact cells; “NC” depicts the cells which were transfected with the negative control ShRNA plasmid; “4′” is clone # 4 in which BRCA1 silencing was optimal one month post ShRNA transfection. “4” is clone # 4 in which BRCA1 silencing was optimal 6 months post ShRNA transfection.

Supplementary Table S1: Statistical parameters of telomere lengths in four chromosomal arms in the BRCA11/2 mutations carriers and controls

	P1		P2		Q1		Q2	
	controls	carriers	controls	carriers	controls	carriers	controls	carriers
Mean	117991	88539	112749	83425	119423	86318	107902	77382
Std. Error	15446	18644	14882	17703	15361	17489	14255	16203
Median	116525	76703	115917	72307	122672	75309	105168	65939
Std. Deviation	46337	58959	44647	55982	46082	55305	42765	51238
Minimum	33928	23041	31698	20061	31854	22270	27632	20287
Maximum	179728	217019	178353	204061	181094	202168	152074	184792
